# Supplementary material for: SOCS1 Deficiency Promotes Hepatocellular Carcinoma via SOCS3-Dependent CDKN1A Induction and NRF2 Activation
Source: Cancers (Basel). 2023 Jan 31;15(3):905. doi: 10.3390/cancers15030905 (PMC9913612; doi:10.3390/cancers15030905)
Supplement: Supplementary file 1 [file cancers-15-00905-s001.zip › cancers-2144517-supplementary.pdf]

SOCS1 Deficiency Promotes Hepatocellular Carcinoma via SOCS3-Dependent CDKN1A Induction and NRF2 Activation

Md Gulam Musawwir Khan et al.,

This PDF file includes:
Supplementary Tables S1 to S5
Supplementary Figures. S1 to S4

Table S1. List of Mouse strains used in this study.

**Strain**	**Citation**	**Supplier**
Socs1$^{fl/fl}$	PMID: 18322180	Dr. Akihiko Yoshimura, Tokyo
Socs3$^{fl/fl}$	PMID: 12754507	Jax mice, Stock # 010944
Alb^{Cre}	PMID: 9867845	Jax mice, Stock # 003574
Cdkn1a$^{-/-}$	PMID: 7566157	Jax mice, Stock # 003263
Tp53$^{-/-}$	PMID: 7922305	Jax mice, Stock # 002101
Socs1$^{fl/fl}$ *Alb*^{Cre}	PMID: 26725321	Previously generated
Socs3$^{fl/fl}$ *Alb*^{Cre}	PMID: 32807134	Previously generated
Socs$^{fl/fl}$ *Socs3*$^{fl/fl}$	PMID: 32807134	Previously generated
Socs$^{fl/fl}$ *Socs3*$^{fl/fl}$ *Alb*^{Cre}		Generated for this study
Cdkn1a$^{-/-}$*Socs1*$^{fl/fl}$ *Alb*^{Cre}		Generated for this study
Tp53$^{-/-}$*Socs1*$^{fl/fl}$ *Alb*^{Cre}		Generated for this study

Table S2. List of reagents used in this study.

**Name**	**Supplier**	**Cat #**
Diethylnitrosamine (DEN)	Sigma-Aldrich	N0258
RNeasy Mini Kit	Qiagen	74104
RNAlater®	Qiagen	76106
QIAzol® Lysis Reagent	Qiagen	79306

Table S3. List of software used.

**Software name**	**Manufacturer**	**Version**
Adobe Photoshop/Illustrator	Adobe Inc.	CC
CFX manager	Bio-Rad Laboratories, Inc	3.1
Image Lab™	Bio-Rad Laboratories, Inc	6
NDP.view2	Hamamatsu Photonics K.K	
Prism	GraphPad	9

Table S4. List of antibodies used in this study.

Name	Citation	Supplier	Cat no.	Clone no.
**Immunofluorescence**				
Rabbit anti- Ki67	PMID: 20152769	Cell Signaling Technology	#9129	D3B5
Mouse anti-4-hydroxy-nonenol (4-HNE)	PMID: 30878614	abcam	Ab48506	HNEJ-2
**Western blot**				
Rabbit-anti p21	PMID: 20959475	Santa Cruz Biotechnology	SC-471	Polyclonal
Mouse-anti p21	PMID: 32499530	Santa Cruz Biotechnology	SC-817	mAb
Rabbit anti-NRF2	PMID: 19417020	Cell Signaling Technology	#12721	D1Z9C
Rabbit anti-Nrf2	PMID: 30896860	abcam	ab156883	Polyclonal
Rabbit anti β-Actin	PMID: 19933848	Cell Signaling Technology	#4970	13E5
Mouse anti-p53	PMID: 26725321	Cell Signaling Technology	#2524	1C12
Rabbit anti-Phospho p53 (Ser15)	PMID: 26725321	Cell Signaling Technology	#9284	Polyclonal
Rabbit anti-Stat3	PMID: 26725321	Santa Cruz Biotechnology	SC-483	Polyclonal
Rabbit anti-Phospho Stat3 (Y705)	PMID: 26725321	Cell Signaling Technology	#9131	Polyclonal

Table S5. List of RT-qPCR primers used in this study.

Gene Name	Gene ID	Forward Sequence (5'-3')	Reverse Sequence (5'-3')	Amplicon size (bp)
36B4 (Rplp0)	NM_007475.5	TCTGGAGGGTGTCCGCAA	CTTGACCTTTTCAGTAAGTGG	154
Cdkn1a	NM_007669.5	TTCTATCACTCCAAGCGCAG	CAGGCAGCGTATATACAGGAG	185
Gadd45a	NM_007836.1	AGTCAGCGCACCATTACG	TGAGGGTGAAATGGATCTGC	139
Gclc	NM_010295.2	ACCATCACTTCATTCCCCAG	TTCTTGTTAGAGTACCGAAGCG	148
Gpx1	NM_008160.6	GACTACACCGAGATGAACGATC	TCTCACCATTCACTTCGCAC	199
Gpx2	NM_030677.2	GTAGTTCTCGGCTTCCCTTG	GGTAAGACTAAAGGTGGGCTG	123
Gstm1	NM_001374678.1	CTATGATACTGGGATACTGGAACG	ACTTCTCATTCAGCCACTGG	147
Gstm4	NM_026764.3	TGAAGGTGGAATACTTGGAGC	GGGTTCGAATATAAGGTGCAGG	149
Hmox1	NM_010442.2	ACAGAGGAACACAAAGACCAG	GTGTCTGGGATGAGCTAGTG	136
Keap1	NM_001110306.1	CTCCGCAGAATGTTACTATCCAG	ACACTGTTCAACTGGTCCTG	147
Mdm2	NM_010786.4	TGGCGTAAGTGAGCATTCTG	CTGTATCGCTTTCTCCTGTCTG	188
Nfe2l2	NM_010902.4	TCCCATTTGTAGATGACCATGAG	CCATGTCCTGCTCTATGCTG	150
Nqo1	NM_008706.5	TGAAGAAGAGAGGATGGGAGG	GATGACTCGGAAGGATACTGAAAG	130
Sesn1	NM_001162908.1	GGACGAGGAACTTGGAATCAG	CAAAGGAGTCTGCAAATAACGC	131
Sesn2	NM_144907.1	CAGCCTCACCTATAACACCATC	CTCGCCGTAATCATAGTCATCG	124
Socs1	NM_001271603.1	TGGTTGTAGCAGCTTGTGTCTGG	CCTGGTTTGTGCAAAGATACTGGG	92
Socs3	NM_007707.3	GAGATTTCGCTTCGGGACTAG	TTTGGAGCTGAAGGTCTTGAG	193
Tp53	NM_011640.3	AGTTCATTGGGACCATCCTG	GCTGATATCCGACTGTGACTC	149

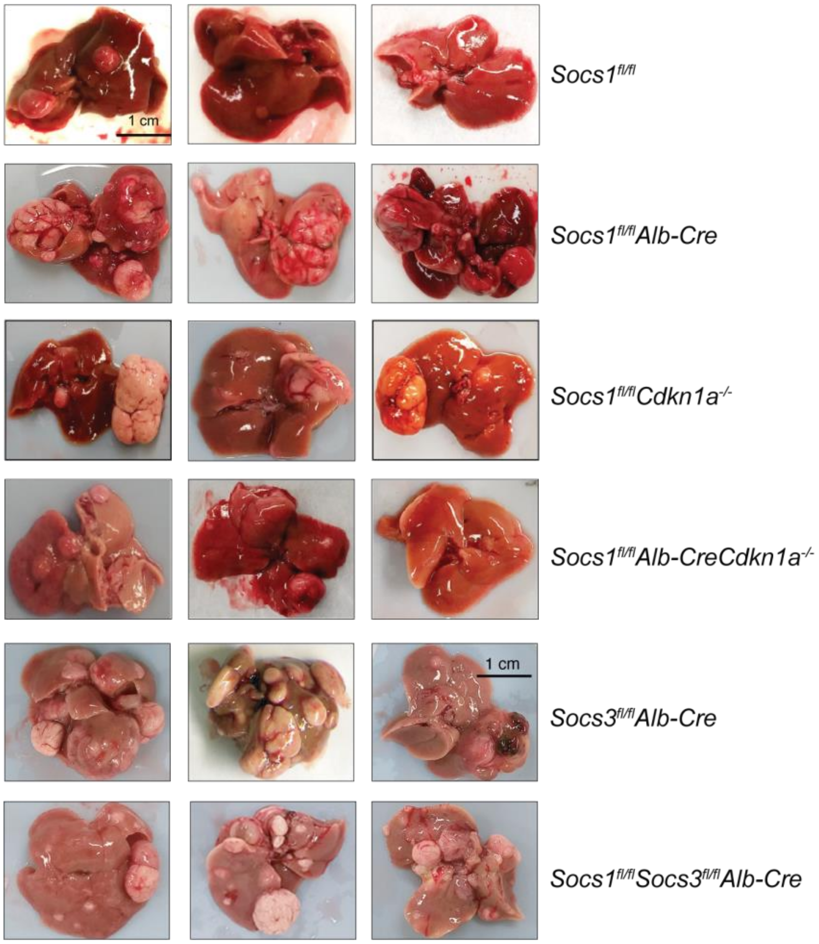

Figure S1. Increased susceptibility of SOCS1-deficient mice to HCC requires CDKN1A and SOCS3. Two weeks-old mice of the indicated genotype were treated with DEN (25 mg/Kg bodyweight, i.p.). HCC development was evaluated ten months later. Livers from three representative mice for each genotype are shown.

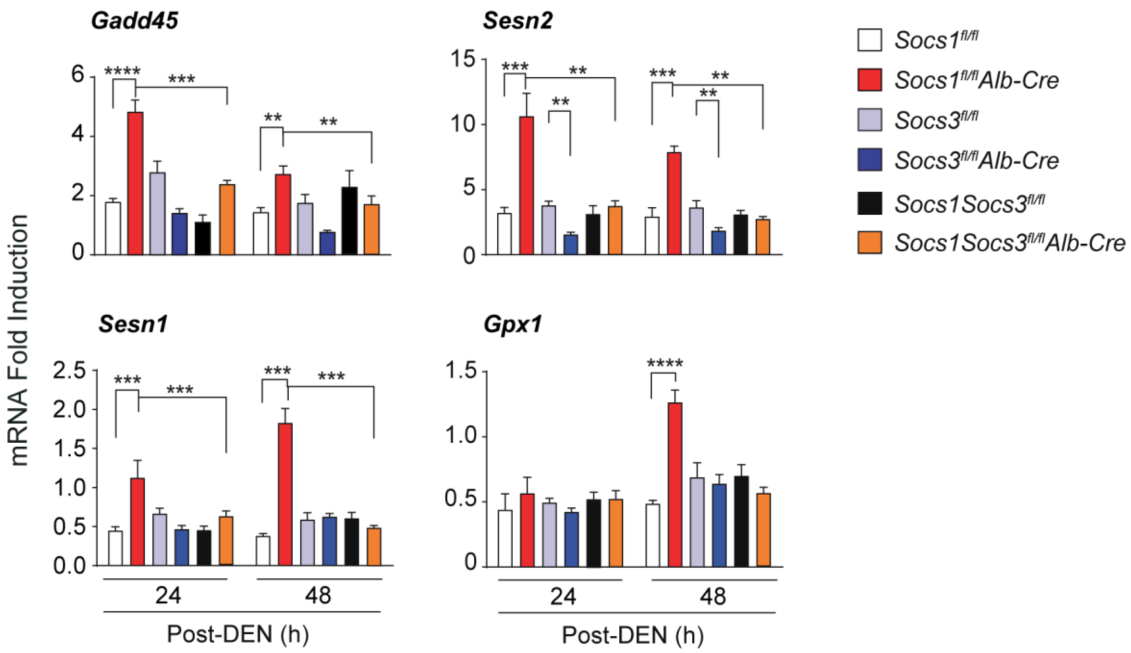

Figure S2. Increased induction of p53 target genes in SOCS1-deficient livers following genotoxic stress requires SOCS3. Mice lacking SOCS1, SOCS3 or both in hepatocytes, and the corresponding control mice were treated with DEN (100 mg/Kg BW, i.p.). Induction of the indicated p53 target genes in the liver tissue was evaluated by RT-qPCR at 24 and 48 h later. n= 3-6 mice per group. mRNA fold induction was calculated relative to the expression in untreated mice of the same genotype after normalization to the housekeeping gene *Rplp0*. (Mean ± SD. Two-way ANOVA with Tukey's test, p: ** <0.01, *** <0.001, **** <0.001.

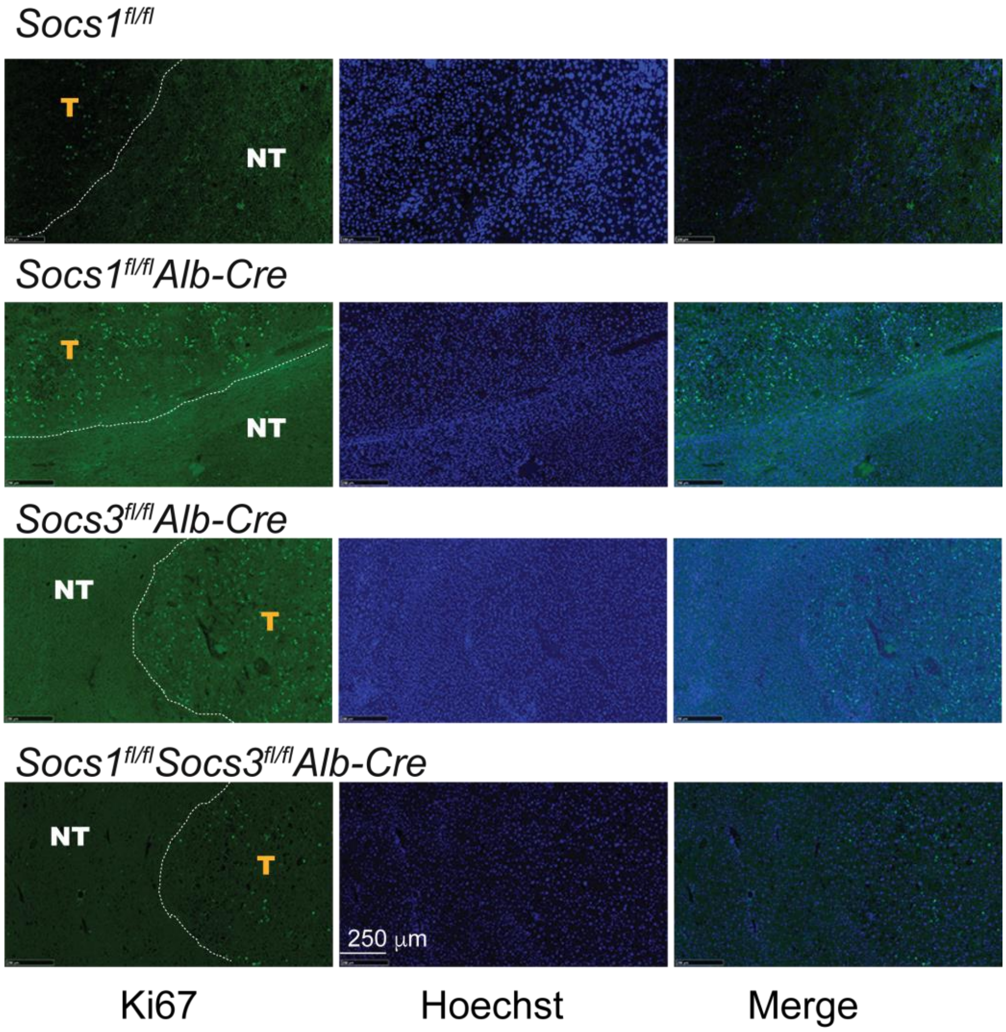

Figure S3. Ki67 immunostaining in DEN-induced HCC. Liver sections from tumor bearing mice of the indicated genotypes (≥3 mice/group) were stained with anti-Ki67 antibody. Representative images for each genotype are shown. Tumor nodules (T) are demarcated from the surrounding normal tissue (NT).

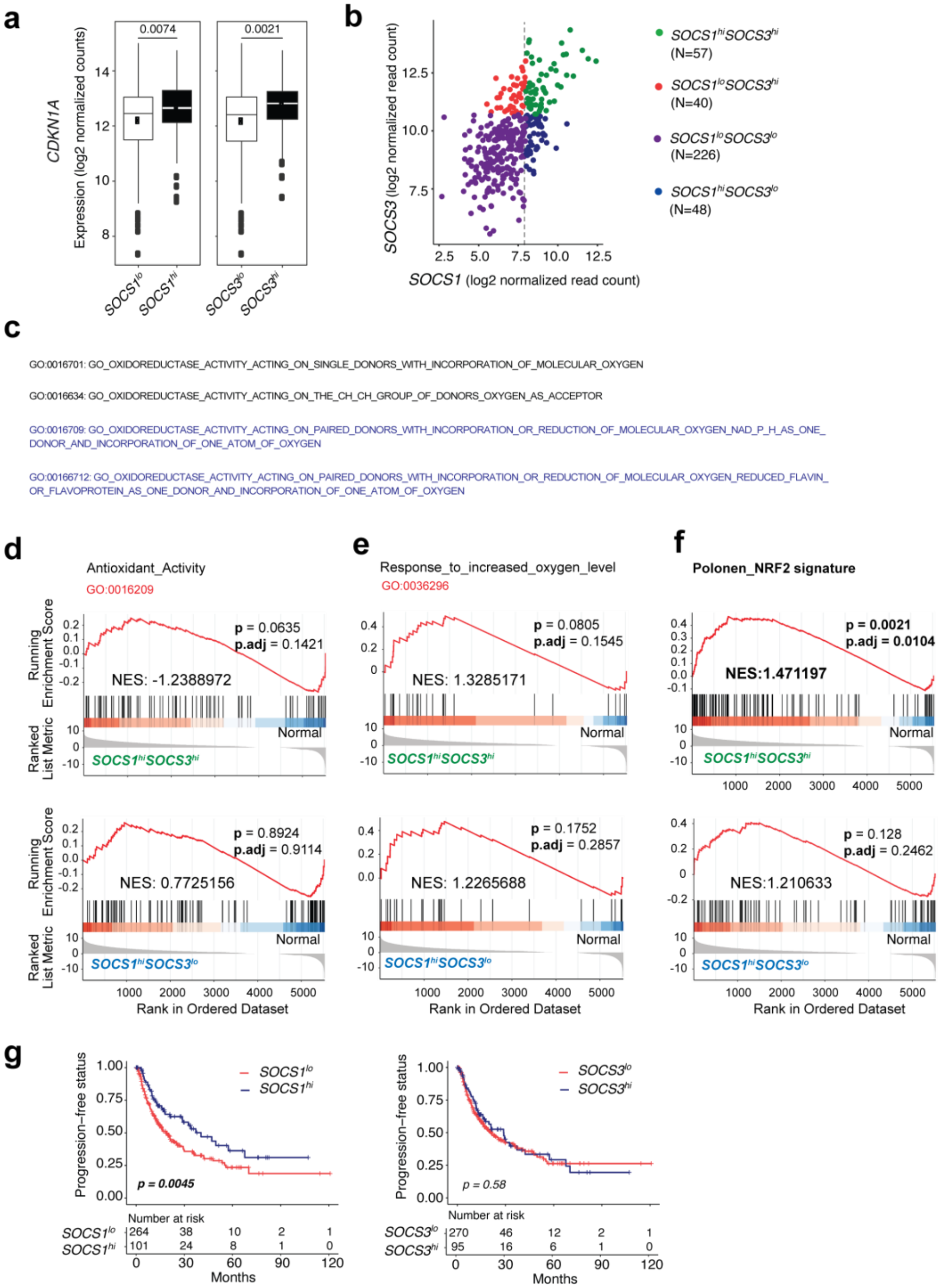

Figure S4. Supplementary data on TCGA-LIHA transcriptomic data analyses. (**a**) *CDKN1A* expression in TCGA-LIHC cases dichotomized based on *SOCS1* or *SOCS3* mRNA levels (z score). (**b**) Segregation of the TCGA-LIHC dataset into *SOCS1-high/SOCS3-high*, *SOCS1-low/SOCS3-high*, *SOCS1-low/SOCS3-low* and *SOCS1-high/SOCS3-low* groups based on z-score. (**c**) GO terms that are too large to expand in Figure 4b. (**d–f**) GSEA plots showing the enrichment levels of the GO terms ‘Antioxidant_Activity’ pathway (**d**) ‘Response_to_increased_oxygen_level’ pathway (**e**) and ‘Polonen_NRF2 Signature’ genes (**f**) in the HCC patient groups not shown in Figure 4. (**g**) Low SOCS1 expression level but not SOCS3 expression predicts poor prognosis.
